# Supplementary material for: Preserved electrophysiological markers of confidence in schizophrenia spectrum disorder
Source: Schizophrenia (Heidelb). 2023 Feb 23;9(1):12. doi: 10.1038/s41537-023-00333-4 (PMC9950441; doi:10.1038/s41537-023-00333-4)
Supplement: Supplementary file 1 — Supplementary Information [file 41537_2023_333_MOESM1_ESM.pdf]

SUPPLEMENTARY INFORMATION

1. Relationship between confidence and confidence history

Consistently with Zheng and colleagues (2022), we applied the following model:

confidence ~ accuracy * group * (RT + confidence history) + (accuracy + RT + conf history| suj)

where confidence history is the confidence averaged over the five trials prior to the current decision.

We found a main effect of RT (Estimate = -0.29, 95%CI [-0.36, -0.23], evidence ratio = 16000), and a main effect of confidence history (Estimate = 0.25, 95%CI [0.18, 0.32], evidence ratio = 16000). We still found an interaction effect between RT and group on confidence level (Estimate = 0.11, 95%CI [0.01, 0.21], evidence ratio = 28.8) indicating that confidence was less correlated with response times among patients compared to control participants. However, there was no interaction between history of confidence and group on confidence (Estimate = 0.04, 95%CI [-0.09, 0.18], BF_{01} = 11.8) indicating that the result obtained by Zheng and colleagues did not extend to our perceptual task (Figure S1).

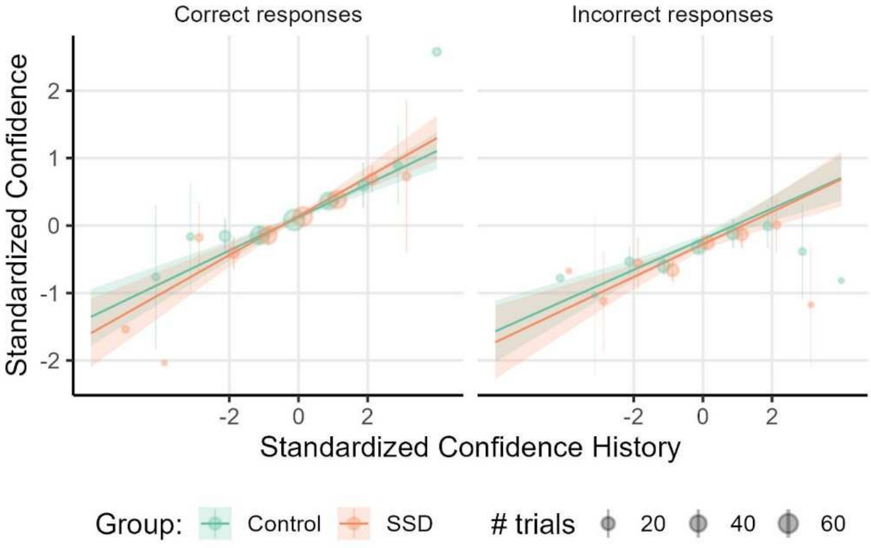

Figure S1: Standardized confidence as a function of standardized confidence history. Dots represent averaged data and lines are regression fits. Control participants are depicted in green, and patients with SSD in red. Error bars represent 95%CI.

2. Time-Frequency analysis

Time-frequency analyses were conducted with the EEGLAB toolbox (v2021.0, EEGLAB, Delorme and Makeig 2004). We used a wavelet decomposition of 165 linearly spaced complex-valued Morlet wavelets ranging from 4 Hz (3 cycles) to 45 Hz (16.875 cycles), For every trial, the EEG signal between -500 ms and 1000 ms after the movement onset was convolved with each Morlet wavelet. We then compared the average magnitude of each condition in the log-domain.

Analysis of confidence for correct trials

Below, we show the time-frequency representation of the confidence contrast between high versus low confidence in correct responses (where high and low categories are determined by a median split of confidence for each participant) for control participants and patients with SSD. We then conducted t-tests to compare the power of each frequency at each time sample between the two groups, while applying False-discovery rate (fdr-) correction for multiple comparisons. No effect of group resisted this correction with a corrected alpha level of 0.05.

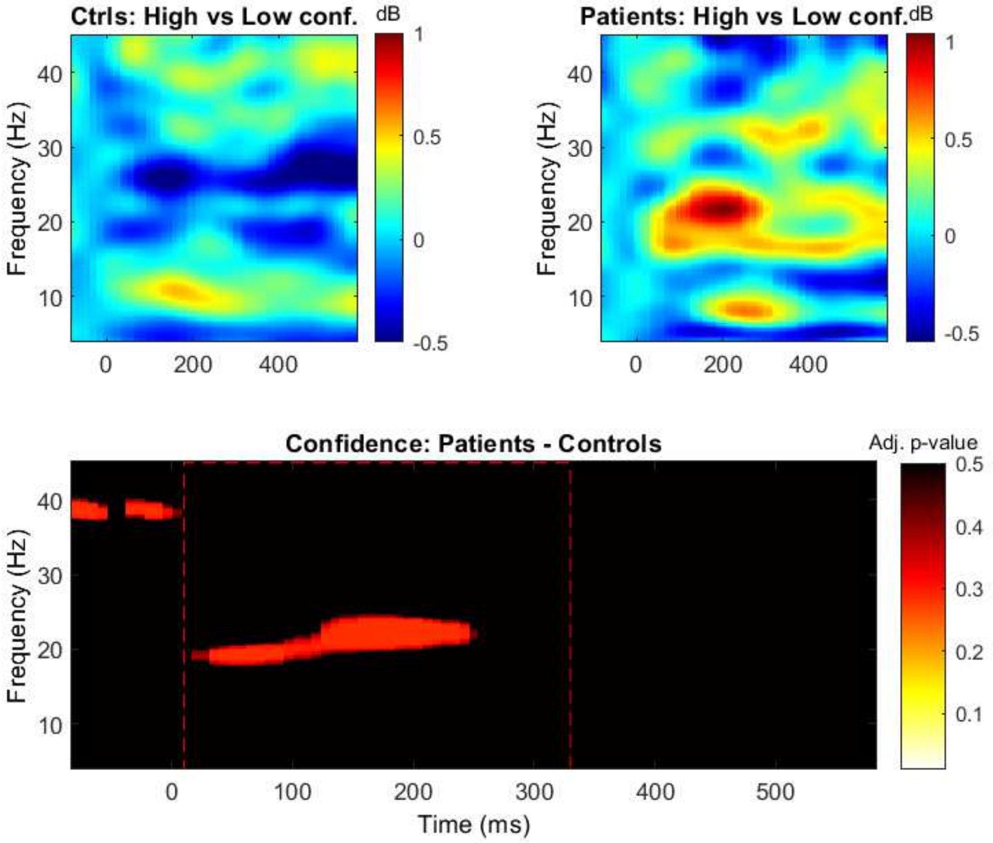

Figure S2: Time-frequency representations. Confidence contrasts (high versus low) for controls (upper left panel) and patients (upper right panel). Diagram of p-values adjusted for multiple comparisons (fdr-correction) between controls and patients (bottom panel). All adjusted p-values are > 0.05. Dashed red lines delimitate the time window where a significant

main effect of confidence on EEG amplitude was found in the cluster analysis reported in the manuscript.

Analysis of correctness

We conducted the same time-frequency analysis for the contrast between correct and incorrect responses for control participants and patients with SSD but again found no significant differences after correcting for multiple comparisons.

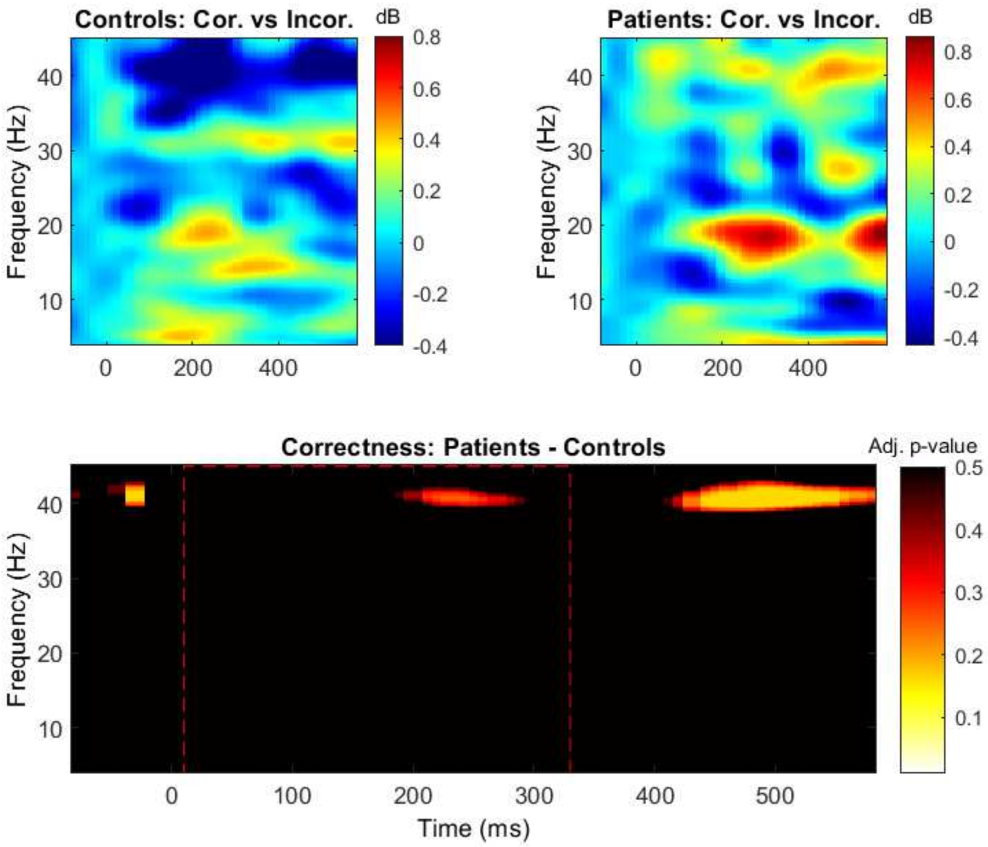

Figure S3: Time-frequency representations. Accuracy contrasts (correct versus incorrect) for controls (upper left panel) and patients (upper right panel). Diagram of adjusted p-values adjusted for multiple comparisons (fdr-correction) between controls and patients (bottom panel). All adjusted p-values are > 0.05. Dashed red lines delimitate the time window where a significant main effect of correctness was found in the cluster analysis reported in the manuscript.
